# Bloodstream infection with NDM-1/5 *Enterobacter cloacae* complex in China: diverse STs, multi-virulence systems and carbapenem resistance

**DOI:** 10.3389/fcimb.2025.1738317

**Published:** 2026-01-14

**Authors:** Xinying Wang, Yujing Tian, Qun Zhang, Yan Jin, Chunhong Shao, Zhijun Zhang

**Affiliations:** 1 Department of Laboratory Medicine, The Affiliated Taian City Central Hospital of Qingdao University, Taian, China; 2 Shandong Provincial Key Medical and Health Laboratory of Anti-Drug Resistant Drug Research, The Affiliated Taian City Central Hospital of Qingdao University, Taian, China; 3 Department of Blood Transfusion, The Affiliated Taian City Central Hospital of Qingdao University, Taian, China; 4 Department of Laboratory Medicine, Shandong Provincial Hospital Affiliated to Shandong First Medical University; Shandong Provincial Hospital, Jinan, China

**Keywords:** *bla*_NDM1/5_, bloodstream infection, carbapenem-resistant, drug resistance, enterobacter cloacae complex, whole-genome sequencing

## Abstract

**Objectives:**

To elucidate the molecular epidemiology, virulence repertoire and resistance gene characteristics of carbapenem-resistant *Enterobacter cloacae* complex (CRECC) in bloodstream infections (BSI), thereby providing evidence for precision therapy and infection control.

**Methods:**

We retrospectively collected 13 non-replicate CRECC-BSI isolates from January 2019 to December 2023 at a tertiary-care hospital in Shandong Province, China. Antimicrobial susceptibility was determined by broth microdilution; Illumina NovaSeq whole-genome sequencing was performed, and genomes were assembled with ABySS and GapCloser. ResFinder, VFDB, CGE and NCBI Pathogen Detection databases were used jointly to analyze resistance genes, virulence factors, plasmid replicons, MLST an SNP-based phylogenetic tree assessed inter-strain relatedness; while filter-mating assays determined the transferability of plasmids.

**Results:**

A total of 13 CRECC isolates yielded five sequence types (STs), with ST171 predominating (46.2%, 6/13); all carried *bla*_NDM_ (*bla*_NDM-1_ in 9 isolates, *bla*_NDM-5_ in 4), along with AmpC, ESBLs, and aminoglycoside/quinolone resistance genes. The IncX3 plasmid replicon was most frequent (46.2%, 6/13), followed by IncHI2/HI2A (38.5%, 5/13). Each strain harbored adherence, biofilm formation, iron/manganese transport and T6SS virulence genes. Antimicrobial susceptibility testing revealed complete resistance among all isolates to cephalosporins, carbapenems and β-lactam/β-lactamase-inhibitor combinations, while amikacin, tigecycline and polymyxin B remained 100% susceptible. cgMLST revealed a polyclonal population structure. Conjugation assays demonstrated transfer of *bla*_NDM_-bearing plasmids to recipient *Escherichia coli* J53.

**Conclusions:**

Our institutional CRECC-BSI is characterized by diverse sequence types, a complex plasmid profile and a high burden of virulence genes; ST171 is the dominant clone and *bla*_NDM-1_ the principal carbapenemase. Close surveillance of this high-risk lineage and of IncX3/IncHI2-mediated horizontal gene transfer is essential, together with strengthened infection-control and antimicrobial-stewardship measures.

## 1 Introduction

The *Enterobacter cloacae* complex (ECC) is a ubiquitous group of opportunistic pathogens that thrive in hospital environments, water and soil, and readily colonize the human gastrointestinal, respiratory and urinary tracts (Wendel et al., 2022). ECC comprises several clinically relevant species, most notably *Enterobacter hormaechei*, *Enterobacter cloacae*, *Enterobacter asburiae* and *Enterobacter roggenkampii* among others (Wu et al., 2025), and can cause a spectrum of healthcare-associated infections including bloodstream, urinary tract, wound, peritonitis and hospital-acquired pneumonia (Emeraud et al., 2024). Bloodstream infection (BSI) is the most severe manifestation, associated with high mortality, prolonged hospitalization and substantial healthcare costs (Gupta et al., 2024; Zhou et al., 2024).

The principal mechanisms of ECC resistance to β-lactam antibiotics are chromosomal AmpC enzyme derepression, and production of extended-spectrum β-lactamases (ESBLs) and carbapenemases (Annavajhala et al., 2019; Yang et al., 2022). These genes can be horizontally transferred via plasmids and integrons, accelerating the spread of resistant clones (Moraz et al., 2021; Fadare and Okoh, 2021). Consequently, the relentless rise of multidrug-resistant (MDR) ECC poses a severe global health threat. Carbapenem-resistant Enterobacterales (CRE) were classified among the pathogens of critical priority by the World Health Organization in 2024 (Antimicrobial Resistance Collaborators, 2022; Miller and Arias, 2024) Nation-wide surveillance underscores this trend: CHINET data show that the prevalence of carbapenem-resistant *Enterobacter cloacae* complex (CRECC) in China rose from 0.7% in 2007 to ~8% in 2024, making it the third most common nosocomial CRE after *Klebsiella pneumoniae* and *Escherichia coli* (Chen et al., 2021; Davin-Regli et al., 2019; China Antimicrobial Surveillance Network, 2024).

Plasmid-mediated New Delhi metallo-β-lactamase (NDM) production is now the dominant resistance mechanism (Bush and Bradford, 2019). NDM enzymes hydrolyze penicillins, cephalosporins and carbapenems, exhibit only weak activity against aztreonam, and remain unaffected by classical β-lactamase inhibitors such as avibactam but are inhibited by EDTA (Nagulapalli Venkata et al., 2021). Consequently, NDM-producing ECC isolates are frequently susceptible only to tigecycline and polymyxins, with occasional aztreonam sensitivity. Epidemiological studies show that healthcare-associated outbreaks of NDM-producing ECC are driven by a restricted set of MLST clones-most notably ST78, ST171 and ST182 (Gomez-Simmonds et al., 2018)-that acquire *bla*_NDM_ on IncX3, IncHI2 or IncFII plasmids (Mavroidi et al., 2023). Once formed, these high-risk ST–plasmid pairings spread rapidly: the ST171-IncX3 complex has driven repeated hospital outbreaks across India, Japan and the United States (Wang et al., 2018), while ST182, tightly associated with IncFII, triggered the largest European ECC epidemic on record in Greece (Gartzonika et al., 2023). Collectively, these findings underscore that the intersection of a susceptible clone with a promiscuous, NDM-carrying replicon acts as a molecular trans-regional escalation switch for ECC, providing clear targets for genomic surveillance.

In China, nationwide molecular epidemiological and virulence data on CRECC-BSI remain scarce. Using whole-genome sequencing and comprehensive in-silico virulence/plasmid profiling, this retrospective, single-center study (n = 13 isolates) systematically delineates the resistance, virulence and transmission patterns of CRECC bloodstream isolates from a tertiary-care hospital in Shandong Province, providing an evidence base for targeted therapy and infection-control strategies.

## 2 Materials and methods

### 2.1 Collection and identification of bacterial isolates

Thirteen non-replicate CRECC isolates first recovered from blood cultures between January 2019 and December 2023 at our hospital were collected. Carbapenem resistance was defined as a MIC ≥ 4 mg/L for imipenem or meropenem (Senneville et al., 2024). Species identification was initially performed using Autof ms1000 (Autobio, China). To achieve species-level resolution within the *Enterobacter cloacae* complex, genome-to-genome distances were calculated against all relevant type strains using the isDDH web server (https://ggdc.dsmz.de/ggdc.php) with GGDC formula 2. A digital DNA-DNA hybridization (dDDH) value of ≥ 70% was considered indicative of the same species.

### 2.2 Antimicrobial susceptibility testing

Antimicrobial susceptibility of all isolates was tested using the VITEK 2 system(bioMérieux, Marcy-l’Étoile, France) against the following agents: cefuroxime (CXM), cefoperazone-sulbactam (SCF), cefotaxime (CTX), ceftazidime (CAZ), cefepime (FEP), aztreonam (ATM), imipenem (IPM), meropenem (MEM), piperacillin-tazobactam (TZP), levofloxacin (LEV), ciprofloxacin (CIP), amikacin (AK), tobramycin (TOB), sulfamethoxazole-trimethoprim (SXT), tigecycline (TGC) and polymyxin B (PB). Antimicrobial susceptibility testing results were interpreted according to the EUCAST (European Committee on Antimicrobial Susceptibility Testing, 2024) criteria for tigecycline and polymyxin B, and the CLSI M100-S34 (Clinical and Laboratory Standards Institute, 2024) criteria for the remaining antibiotics. *Escherichia coli* ATCC 25922 served as the quality-control strain.

### 2.3 Whole-genome sequencing and bioinformatics analysis

Fresh CRECC colonies from blood agar were selected and high-quality genomic DNA extracted using the OMEGA Bacterial DNA Kit (Omega Bio-tek, Inc., Norcross, GA, USA) following the manufacturer’s instructions. DNA was sent to Shanghai Baizelong Biotechnology Co., Ltd. for library preparation and sequencing on the Illumina NovaSeq platform. Reads were quality-trimmed and assembled with ABySS using a multi-k-mer strategy; gaps were closed and single-nucleotide errors corrected with GapCloser. Resistance genes were identified using ResFinder (http://www.genomicepidemiology.org/services/). Plasmid replicons were extracted via BAcWGSTdb, and virulence factors were screened with VFAnalyst (http://www.mgc.ac.cn/VFs/, accessed).

### 2.4 Multilocus sequence typing and phylogenetic analysis

Seven housekeeping loci (*dnaA, fusA, gyrB, leuS, pyrG, rplB and rpoB*) were extracted (Miyoshi-Akiyama et al., 2013) and queried against the *Enterobacter cloacae* PubMLST database to assign sequence types (STs). For phylogenomic analyses, 41 publicly available CRECC blood isolates from mainland China were downloaded from NCBI Pathogen Detection (http://www.ncbi.nlm.nih.gov/pathogens/); core-genome SNPs were called with CSI Phylogeny (CGE, DTU) - chosen for its robust performance within the Enterobacteriaceae MLST framework and previously validated reliability on ECC genomes and a phylogenetic tree was constructed. The final phylogenetic tree was visualized and annotated using ChiPlot (Xie et al., 2023).

### 2.5 Conjugation assay

Donor CRECC and sodium-azide-resistant *E. coli* J53 were mixed 1:1 (500 µl each), filter-mated for 4 h, and plated on Mueller–Hinton agar containing IPM (4 mg/L) plus sodium azide (50 mg/L). Transconjugants were retained only when they met all three criteria: (1) IPM or MEM MIC ≥ 2 mg/L; (2) tolerance to sodium azide; and (3) sequencing-positive for *bla*_NDM_.

## 3 Results

### 3.1 Clinical characteristics

From January 2019 to December 2023, 13 patients with CRECC-BSI were enrolled. Eleven (84.6%) were male and two (15.4%) female; the median age was 63 years (range 2-91). Nine patients (69.2%) had been admitted to the ICU, 11 (84.6%) had undergone invasive procedures such as central-venous catheterization, mechanical ventilation or indwelling urinary catheterization, and eight (61.5%) had received carbapenems (defined as ≥ 48 h intravenous therapy before the first positive blood culture) prior to the positive blood culture. The presumed sources of infection were pulmonary (n = 5), urinary tract (n = 3) and unknown (n = 5) (**Table 1**).

**Table 1:** Baseline and clinical characteristics of 13 patients with CRECC bloodstream infection, 2019–2023.

Patient ID	Year	Age (y)	Sex	Ward	Antimicrobial exposure prior to CRECC detection	Antimicrobial therapy after CRECC detection	Length of stay (d)	Source of BSI	Invasive procedures	ICU Admission	Outcome
CRECC17	2019	43	M	ICU	P, LEV, MEM, VA	SCF, TGC	36	Pulmonary	UC, MV	Yes	Survived
CRECC32	2020	2	M	Hematology	IPM, LZD	TGC, AK, IPM	20	Unknown	CVC	No	Survived
CRECC36	2021	71	M	EICU	TZP, MEM, TGC, SCF, CAZ, LZD, AK	TGC, MEM, LZD, LEV, ATM, CZA, CIP	188	Pulmonary	UC, CVC, MV	Yes	Survived
CRECC39	2021	67	F	Hematology	LZD, IPM, MEM	SCF, TGC, ATM	21	Pulmonary	None	No	Survived
CRECC44	2021	54	F	NICU	OX, CIP, TZP	IPM, ATM, FOS	66	Unknown	UC, CVC	Yes	Survived
CRECC54	2021	88	M	ICU	LEV	CAZ, MEM, IPM	171	Pulmonary	UC, CVC	Yes	Survived
CRECC60	2022	58	M	RICU	MEM	MEM, TGC	25	Unknown	UC, CVC, MV	Yes	Survived
CRECC61	2022	69	M	ICU	MEM, CXM, LZD	MEM, LZD, PB, SCF, TGC, FEP	96	Unknown	UC, CVC, MV	Yes	Survived
CRECC76	2022	57	M	RICU	TZP	SCF, TGC	55	Catheter-related	UC, CVC, MV	Yes	Died
CRECC77	2022	55	M	Neurology	TZP, MFX	IPM, AK	15	Urinary	UC	No	Survived
CRECC110	2023	63	M	Hematology	MFX, MEM, SCF, TGC	ATM, IPM	24	Pulmonary	None	No	Died
CRECC117	2023	86	M	ICU	FOS, MEM	VA, MEM, FOS, TZP, TGC, PB, LZD	187	Urinary	UC, CVC, MV	Yes	Died
CRECC118	2023	91	M	CICU	SCF	ATM, FOS, TZP	19	Urinary	UC	Yes	Survived

AK, amikacin; ATM, aztreonam; CAZ, ceftazidime; CXM, cefuroxime; CZA, ceftazidime–avibactam; CIP, ciprofloxacin; EICU, emergency intensive care unit; FEP, cefepime; FOS, fosfomycin; IPM, imipenem; LEV, levofloxacin; LZD, linezolid; MFX, moxifloxacin; MEM, meropenem; MV, mechanical ventilation; NICU, neonatal intensive care unit; OX, oxacillin; PB, polymyxin B; RICU, respiratory intensive care unit; SCF, cefoperazone–sulbactam; TZP, piperacillin–tazobactam; TGC, tigecycline; UC, indwelling urinary catheter; VA, vancomycin, BSI, bloodstream infection; ICU, intensive care unit; CVC, central venous catheter; CRECC, Carbapenem-Resistant *Enterobacter cloacae* complex.

### 3.2 Species identification and phylogeny analysis

**Figure 1:**
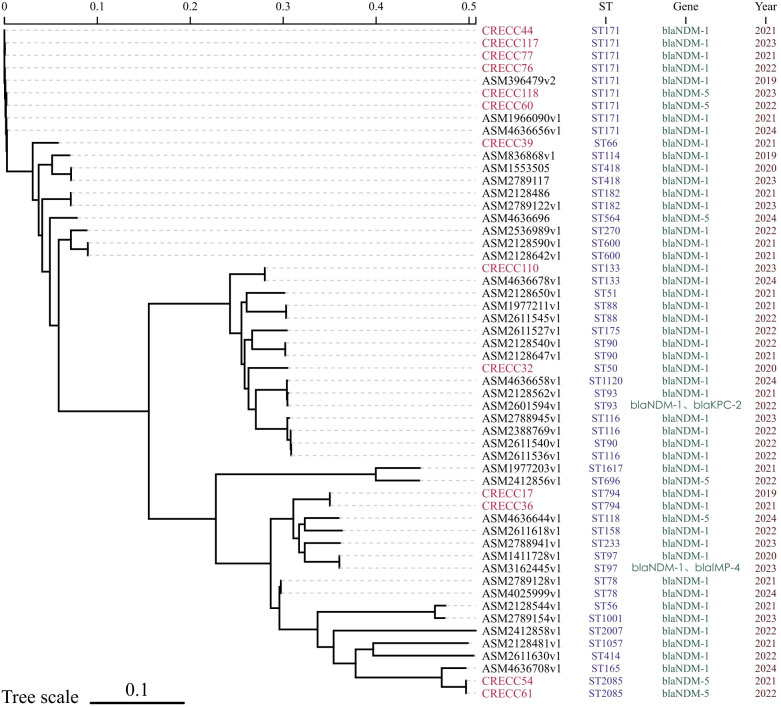
Phylogenetictreeof 54 CRECC strains. Red color denotes isolates obtained in our hospital.

MALDI-TOF MS accurately assigned all 13 isolates to the *Enterobacter cloacae* complex (100% concordance at the complex level).However, at the species level, only 4/13 designations were confirmed by the genome-based digital DNA-DNA hybridization (dDDH) analysis (30.8% concordance), demonstrating the limited resolution of MALDI-TOF within this complex (**Supplementary Table S1**) MLST revealed five sequence types: ST171 predominated (6/13, 46.2%), followed by ST794 and ST2085 (2/13 each, 15.4%), and ST50, ST66 and ST133 (1 isolate each). When combined with 41 bloodstream CRECC sequences downloaded from NCBI, ST171 remained the most common type nationwide (9/54) and all carried *bla*_NDM-1_ (**Figure 1**). cgMLST uncovered the genetic divergence among strains of the ST171 clone : pairwise core-genome SNP distances ranged from 4 to 63 (median 11) (**Supplementary Table S2**).

### 3.3 Resistance genes, phenotypes and conjugation analysis

**Figure 2:**
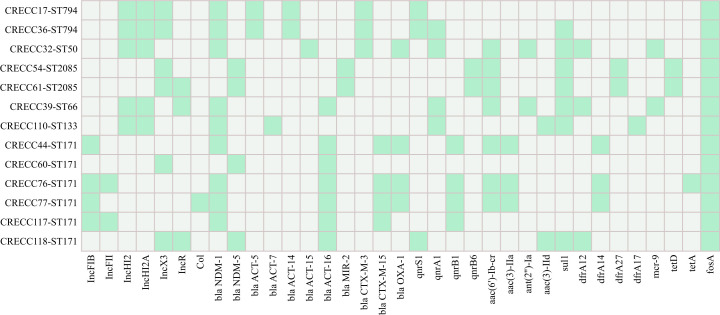
Heat-map displaying plasmid replicon profiles (left panel) and resistance-gene content (right panel) of 13 CRECC bloodstream isolates. X-axis represents plasmid replicons and resistance genes, Y-axis shows the strain sequence numbers, Green fill indicates presence of the corresponding gene; Grey fill indicates absence.

Whole-genome analysis revealed that all isolates carried *bla*_NDM_: *bla*_NDM-1_ in 9 isolates (69.2%) and *bla*_NDM-5_ in 4 (30.8%). Additional resistance genes were detected at the following frequencies: *bla*_ACT-16_ (53.8%), *bla*_CTX-M-15_ (30.8%), *bla*_CTX-M-3_ (23.1%), *bla*_OXA-1_ (30.8%), *aac(6′)-Ib-cr* (53.8%), *aac(3)-II* (23.1%), *qnrB1/qnrA1* (each 30.8%), *sul1* (53.8%) and *mcr-9* (15.4%) (**Figure 2**). Antimicrobial susceptibility testing showed that all 13 isolates were resistant to cephalosporins, carbapenems and β-lactam/β-lactamase-inhibitor combinations; resistance rates to LEV, CIP, TO, SXT and ATM were 84.6%, 92.3%, 61.5%, 53.8% and 46.2%, respectively. AK, TGC and PB retained 100% susceptibility (**Table 2**). In filter-mating assays using the 13 CRECC isolates as donors and *E. coli* J53 as recipient, five (38.5%) successfully transferred *bla*_NDM_, yielding transconjugants CRECC44, 54, 61, 76 and 77. Post-conjugation, recipient MICs rose from ≤ 0.25 mg/L to 2–≥ 16 mg/L for IPM and ≥ 16 mg/L for MEM, and sequencing confirmed the presence of the same *bla*_NDM_ subtype as the donor (**Table 2**).

**Table 2:** MICs and sequence types of 13 CRECC clinical isolates and five transconjugants against 16 antimicrobial agents.

Isolation ID	Carbapenemase-encoding genes	ST type	CXM	SCF	CTX	CAZ	FEP	ATM	IPM	MEM	TZP	LEV	CIP	AK	TOB	SXT	PB	TGC
CRECC17	NDM-1	794	≥64	≥64	≥64	≥64	≥32	4	≥16	≥16	≥128/4	≥8	≥4	≤2	≤1	≤1/19	≤0.5	2
CRECC32	NDM-1	50	≥64	≥64	≥64	≥64	≥32	4	≥16	≥16	≥128/4	0.5	0.25	≤2	8	>4/76	≤0.5	≤0.5
CRECC36	NDM-1	794	≥64	≥64	≥64	≥64	≥32	4	≥16	≥16	≥128/4	≥8	≥4	≤2	<=1	>4/76	≤0.5	2
CRECC39	NDM-1	66	≥64	≥64	≥64	≥64	≥32	≤1	≥16	≥16	≥128/4	1	1	≤2	8	>4/76	≤0.5	1
CRECC44	NDM-1	171	≥64	≥64	≥64	≥64	≥32	≥64	≥16	≥16	≥128/4	≥8	≥4	8	≥16	≤1/19	≤0.5	1
CRECC54	NDM-5	2085	≥64	≥64	≥64	≥64	≥32	16	≥16	≥16	≥128/4	≥8	≥4	≤2	8	>4/76	≤0.5	≤2
CRECC60	NDM-5	171	≥64	≥64	≥64	≥64	16	≤1	≥16	≥16	≥128/4	4	≥4	≤2	≤1	≤1/19	≤0.5	2
CRECC61	NDM-5	2085	≥64	≥64	≥64	≥64	≥32	16	≥16	≥16	≥128/4	≥8	≥4	≤2	8	>4/76	≤0.5	≤2
CRECC76	NDM-1	171	≥64	≥64	≥64	≥64	≥32	≥64	≥16	≥16	≥128/4	≥8	≥4	4	≥16	≤1/19	≤0.5	1
CRECC77	NDM-1	171	≥64	≥64	≥64	≥64	≥32	≥64	≥16	≥16	≥128/4	≥8	≥4	8	≥16	≤1/19	≤0.5	2
CRECC110	NDM-1	133	≥64	≥64	≥64	≥64	≥32	≤1	≥16	≥16	≥128/4	2	1	≤2	4	>4/76	≤0.5	≤2
CRECC117	NDM-1	171	≥64	≥64	≥64	≥64	≥32	≥64	≥16	≥16	≥128/4	≥8	≥4	≤2	1	≤1/19	≤0.5	1
CRECC118	NDM-5	171	≥64	≥64	≥64	≥64	≥32	≤1	≥16	≥16	≥128/4	≥8	≥4	≤2	8	>4/76	≤0.5	≤2
CRECC44TC	NDM-1	–	≥64	≥64	≥64	≥64	2	≤1	2	≥16	≥128/4	≤0.12	≤0.25	≤2	≤1	≤1/19	≤0.5	≤0.5
CRECC54TC	NDM-5	–	≥64	≥64	≥64	≥64	8	≤1	≥16	≥16	≥128/4	≤0.12	≤0.25	≤2	≤1	≤1/19	≤0.5	≤0.5
CRECC61TC	NDM-5	–	≥64	≥64	≥64	≥64	16	≤1	≥16	≥16	≥128/4	≤0.12	≤0.25	≤2	≤1	≤1/19	≤0.5	≤0.5
CRECC76TC	NDM-1	–	≥64	≥64	≥64	≥64	4	≤1	8	≥16	≥128/4	≤0.12	≤0.25	≤2	≤1	≤1/19	≤0.5	≤0.5
CRECC77TC	NDM-1	–	≥64	≥64	≥64	≥64	2	≤1	8	≥16	≥128/4	≤0.12	≤0.25	≤2	≤1	≤1/19	≤0.5	≤0.5
J53	–	–	4	≤8	≤1	≤0.12	≤0.12	≤1	≤0.25	≤0.25	≤4/4	≤0.12	≤0.25	≤2	≤1	≤1/19	≤0.5	≤0.5

Grey-shaded were interpreted as resistant; CXM, cefuroxime; SCF, cefoperazone/sulbactam; CTX, cefotaxime; CAZ, ceftazidime; FEP, cefepime; ATM, azlocillin; IPM, imipenem; MEM, meropenem; TZP, piperacillin/tazobactam; LEV, levofloxacin; CIP, ciprofloxacin; AK, amikacin; TOB, tobramycin; SXT, sulfamethoxazole-trimethoprim; TGC, tigecycline; PB, polymyxin B; TC, transconjugant strain.

### 3.4 Plasmid replicons and virulence profiles

IncX3 was the most prevalent plasmid replicon (6/13, 46.2%), followed by IncHI2/HI2A (5/13, 38.5%) and IncR (4/13, 30.8%), with three isolates co-harboring IncX3+IncR (**Supplementary Table S1**). Among the five successful transconjugants, a single replicon was consistently retained: IncFII for *bla*_NDM-1_ (ST171) and IncX3 for *bla*_NDM-5_ (ST2085). For virulence systems, all strains carried adherence, *iron*/manganese transport, biofilm formation and T6SS modules; isolates CRECC76, 77, 110 and 117 harbored the complete set of screened virulence genes (**Table 3**).

**Table 3:** Distribution of virulence-associated genes among 13 CRECC isolates.

VF classs	Relate genes	CRECC17	CRECC32	CRECC36	CRECC39	CRECC44	CRECC54	CRECC60	CRECC61	CRECC76	CRECC77	CRECC110	CRECC117	CRECC118
		ST794	ST50	ST794	ST66	ST171	ST2085	ST171	ST2085	ST171	ST171	ST133	ST171	ST171
Adherence														
Curli fibers	*csgC*													
P fimbriae	*papC*													
Type I fimbriae	*fimACD*													
Autotransporter	*ehaB*													
Invasion														
Invasion of brain endothelial cells (Ibes)	*ibeB*													
Flagella(Burkholderia)	*cheW*													
Iron uptake														
Aerobactin siderophore	*iucABCD,iutA*													
Heme uptake	*chuASU*													
Iron/manganese transport	*sitABCD*													
Salmochelin siderophore	*iroN*													
Regulation	*aggR*													
Secretion system														
SCI-I T6SS	*--*													
T2SS	*--*													
Toxin	*ast*													
Antiphagocytosis														
Capsular polysaccharide(Vibrio)	*wzb*													
Biofilm formation	*adeG、pgaC*													
Immune evasion	*gtrA*													
Serum resistance	*--*													
Stress adaptation	*mntB*													
Endotoxin	*HtrBF,wbaP/rfbP*													

Green indicates the presence of the gene; yellow indicates its absence.

## 4 Discussion

BSI is a life-threatening condition caused by the invasion of pathogenic microorganisms into the circulation and remains one of the most severe complications in clinical practice. Its high mortality, coupled with the global spread of multidrug-resistant organisms, poses formidable challenges to public health (Temkin et al., 2024). Recent diagnostic and therapeutic advances have lagged behind the rapid emergence of resistant pathogens, underscoring the need for improved prevention and management strategies. CRECC-BSI is closely linked to prior CRECC colonization or infection, extensive antibiotic exposure (especially carbapenems) and invasive procedures that disrupt physiological barriers (Lumbreras-Iglesias et al., 2023). In this retrospective cohort of 13 patients, 84.6% were male, 84.6% underwent invasive interventions, and 62.9% were admitted to the ICU; these figures that align with the Italian multicenter CR-GNB BSI report (Falcone et al., 2023). Pulmonary (38.5%) and urinary-tract (23.1%) sources predominated, indicating that airway- and catheter-related infections are the main portals of entry in our hospital. Notably, 61.5% of patients had received carbapenems for ≥48 h before blood cultures became positive, a selective pressure that favors resistant clones.

In our results, all 13 isolates carried *bla*NDM (NDM-1 69.2%, NDM-5 30.8%); their geographical distribution differs markedly from the KPC predominance in North America and the VIM/OXA-48 prevalence in Europe, but aligns with the ongoing NDM surge reported in southern, north-eastern and north-western China (Chen et al., 2021; Han et al., 2023; Cai et al., 2024; Shi et al., 2017; Tian et al., 2020), underscoring pronounced regional divergence of CRECC resistance. In addition to carbapenemases, each isolate carried an AmpC β-lactamase of distinct subtype (ACT-16; n = 7, ACT-14; n = 2, MIR-2; n = 2, ACT-15; n = 1, ACT-5; n = 1, ACT-7; n = 1), while 50% harbored ESBLs (CTX-M-15 30.8%, CTX-M-3 23.1%), consistent with a previous report by Cai et al (Cai et al., 2024). Detection of two *mcr-9*-positive isolates further restricts polymyxin options and complicates therapeutic choices.

MLST revealed that ST171 accounted for 46.2% of isolates; in-hospital deaths were associated with this lineage, consistent with US and Japanese reports that designate ST171 as a hyper-virulent, highly transmissible clone (Pereira et al., 2019; Harada et al., 2017; Ghazawi et al., 2024). Although three isolate pairs differed by ≤5 SNPs and may represent short-term transmission links (Snitkin et al., 2012), the majority showed >20 SNP differences, indicating absence of a single-strain nosocomial outbreak. ST93, frequently reported in China, was not detected (Zhao et al., 2020). NCBI data reveal that Chinese bloodstream CRECC isolates exhibit diverse, sporadic STs, mirroring the polyclonal pattern observed here (Gomez-Simmonds et al., 2016). Based on the results of the conjugation assays, five donor strains harboring *bla*_NDM_ successfully transferred their resistance plasmids to recipient *E. coli* J53, yielding transconjugants with ≥8-fold MIC increases for IPM and MEM compared with the recipient. Despite donors harboring multiple replicons, every transconjugant carried only a single plasmid replicon. IncFII for *bla*_NDM-1_ (ST171) or IncX3 for *bla*_NDM-5_ (ST2085). These results confirm the superior conjugative fitness of both replicons. They also align with global surveillance data identifying IncFII and IncX3 as the primary vehicles for *bla*_NDM-1_ and *bla*_NDM-5_ dissemination, respectively (Carattoli, 2013; Mouftah et al., 2019). These data provide direct experimental evidence that plasmid-mediated carbapenem resistance can be functionally transferred within our hospital environment.

Our results shown every isolate harbored the type VI secretion system (T6SS), a key virulence module that directly contributes to bacterial pathogenicity and host interaction, thereby posing a major clinical threat (Ghazawi et al., 2024; Theriault et al., 2021). Additionally, the adherence system was detected at varying levels; by promoting cell adhesion and interacting with the host immune response, it amplifies inflammatory reactions. The diverse virulence repertoire of CRECC strains thus complicates both the treatment and control of bloodstream infections (Azam et al., 2023). The 13 CRECC isolates harbored multiple plasmid replicon types, with IncHI2, IncHI2A and IncX3 being the most prevalent — mirroring reports from North America and Europe (Wu et al., 2025). IncX3 was detected most frequently, in line with a recent nationwide survey in China; its small size and high stability facilitate rapid inter-strain transfer of resistance genes (Wang et al., 2018). These findings underscore the need for early rectal screening of carbapenem-resistant organisms in high-risk patients, minimization of invasive procedures, prompt device replacement with regular microbiological surveillance, and enhancement of host immunity to curtail the spread of CRECC.

## 5 Conclusion

Our institutional CRECC-BSI isolates exhibited diverse sequence types, a complex plasmid repertoire and multiple virulence genes. ST171 was the dominant clone, NDM-1 the principal carbapenemase, and IncX3/IncHI2 plasmids the key vehicles for resistance-gene dissemination. Enhanced surveillance of this high-risk lineage and interventions against plasmid-mediated horizontal transfer are essential to curb further spread of CRECC in health-care settings.

## 6 Limitations

This study has several limitations. First, its retrospective, single-center design may introduce selection bias and limit the generalizability of our findings. Second, the relatively small sample size may not fully represent the national molecular epidemiology of CRECC. Future work should enlarge the isolate collection and integrate multicenter prospective data to obtain a comprehensive picture of CRECC trends and resistance mechanisms.

## Data Availability

The datasets presented in this study can be found in online repositories. The names of the repository/repositories and accession number(s) can be found in the article/**Supplementary Material**.
