# Supplementary material for: Bloodstream infection with NDM-1/5 Enterobacter cloacae complex in China: diverse STs, multi-virulence systems and carbapenem resistance
Source: Front Cell Infect Microbiol. 2026 Jan 14;15:1738317. doi: 10.3389/fcimb.2025.1738317 (PMC12847444; doi:10.3389/fcimb.2025.1738317)
Supplement: Supplementary file 1 [file Table1.doc]

Table S1. Species designation based on dDDH analysis and plasmid profiles of the 13 CRECC isolates.

| **Isolate** | **Autof ms1000 initial call** | **ST type** | **Plasmid replicon**  **(s)** | **ANI-based species** | **dDDH**  **（%）** | **Reference strain** |
| --- | --- | --- | --- | --- | --- | --- |
| CRECC17 | *Enterobacter cloacae* | 794 | IncHI2,IncHI2A, IncX3 | *Enterobacter hormaechei* | 82.8 | NZ_CP017186.1 |
| CRECC32 | *Enterobacter hormaechei* | 50 | IncHI2, IncHI2A | *Enterobacter hormaechei* | 79.6 | NZ_CP017179.1 |
| CRECC36 | *Enterobacter cloacae* | 794 | IncHI2, IncHI2A, IncX3 | *Enterobacter hormaechei* | 83.7 | NZ_CP017186.1 |
| CRECC39 | *Enterobacter hormaechei* | 66 | IncHI2, IncHI2A, IncR | *Enterobacter hormaechei* | 85.3 | NZ_CP017183.1 |
| CRECC44 | *Enterobacter cloacae* | 171 | IncFIB | *Enterobacter hormaechei* | 85.8 | NZ_CP017183.1 |
| CRECC54 | *Enterobacter cloacae* | 2085 | IncR, IncX3 | *Enterobacter roggenkampii* | 90.4 | NZ_CP017184.1 |
| CRECC60 | *Enterobacter cloacae* | 171 | IncX3 | *Enterobacter hormaechei* | 70.8 | NZ_CP017183.1 |
| CRECC61 | *Enterobacter asburiae* | 2085 | IncR, IncX3 | *Enterobacter roggenkampii* | 90.3 | NZ_CP017184.1 |
| CRECC76 | *Enterobacter hormaechei* | 171 | Col, IncFIB, IncFII | *Enterobacter hormaechei* | 86.6 | NZ_CP017183.1 |
| CRECC77 | *Enterobacter cloacae* | 171 | IncFIB | *Enterobacter hormaechei* | 86.4 | NZ_CP017183.1 |
| CRECC110 | *Enterobacter cloacae* | 133 | IncHI2, IncHI2A | *Enterobacter hormaechei* | 82.2 | NZ_CP017179.1 |
| CRECC117 | *Enterobacter cloacae* | 171 | IncFIB, IncFII | *Enterobacter hormaechei* | 86.5 | NZ_CP017183.1 |
| CRECC118 | *Enterobacter hormaechei* | 171 | IncR, IncX3 | *Enterobacter hormaechei* | 88.4 | NZ_CP017183.1 |

dDDH, digital DNA-DNA hybridization
