# Supplementary material for: Bloodstream infection with NDM-1/5 Enterobacter cloacae complex in China: diverse STs, multi-virulence systems and carbapenem resistance
Source: Front Cell Infect Microbiol. 2026 Jan 14;15:1738317. doi: 10.3389/fcimb.2025.1738317 (PMC12847444; doi:10.3389/fcimb.2025.1738317)
Supplement: Supplementary file 2 [file Table2.doc]

Table S2. Pairwise core-genome SNP differences among ST171 isolates (n = 6)

| Isolates | CRECC117 | CRECC118 | CRECC44 | CRECC60 | CRECC76 | CRECC77 |
| --- | --- | --- | --- | --- | --- | --- |
| CRECC117 | 0 | 39 | **5** | 58 | 11 | **4** |
| CRECC118 | 39 | 0 | 34 | 45 | 42 | 39 |
| CRECC44 | **5** | 34 | 0 | 57 | 12 | **5** |
| CRECC60 | 58 | 45 | 57 | 0 | 63 | 58 |
| CRECC76 | 11 | 42 | 12 | 63 | 0 | 11 |
| CRECC77 | **4** | 39 | **5** | 58 | 11 | 0 |

Diagonal cells = 0 (self-comparison). Values ≤10 SNPs (potential recent transmission pairs) are shown in bold.
