# Supplementary material for: Bloodstream infection with NDM-1/5 Enterobacter cloacae complex in China: diverse STs, multi-virulence systems and carbapenem resistance
Source: Front Cell Infect Microbiol. 2026 Jan 14;15:1738317. doi: 10.3389/fcimb.2025.1738317 (PMC12847444; doi:10.3389/fcimb.2025.1738317)
Supplement: Supplementary file 3 [file Table3.doc]

Table S3. BioSample accession numbers and final species designations of the 13 CRECC isolates.

| **Strain ID** | **BioSample Accession** | **Organism** |
| --- | --- | --- |
| CRECC17 | SAMN53056203 | *Enterobacter hormaechei* |
| CRECC32 | SAMN53056204 | *Enterobacter hormaechei* |
| CRECC36 | SAMN53056205 | *Enterobacter hormaechei* |
| CRECC39 | SAMN53056206 | *Enterobacter hormaechei* |
| CRECC44 | SAMN53056207 | *Enterobacter hormaechei* |
| CRECC54 | SAMN53056208 | *Enterobacter roggenkampii* |
| CRECC60 | SAMN53056209 | *Enterobacter hormaechei* |
| CRECC61 | SAMN53056210 | *Enterobacter roggenkampii* |
| CRECC76 | SAMN53056211 | *Enterobacter hormaechei* |
| CRECC77 | SAMN53056212 | *Enterobacter hormaechei* |
| CRECC110 | SAMN53056213 | *Enterobacter hormaechei* |
| CRECC117 | SAMN53056214 | *Enterobacter hormaechei* |
| CRECC118 | SAMN53056215 | *Enterobacter hormaechei* |
